# Isolation of esophageal stem cells with potential for therapy

**DOI:** 10.1007/s00383-014-3615-6

**Published:** 2014-10-30

**Authors:** Panagiotis Maghsoudlou, Daniel Ditchfield, Dorota H. K. Klepacka, Panicos Shangaris, Luca Urbani, Stavros P. Loukogeorgakis, Simon Eaton, Paolo De Coppi

**Affiliations:** Stem Cells and Regenerative Medicine Section, UCL Institute of Child Health and Surgery Unit, Great Ormond Street Hospital, 30 Guilford Street, London, WC1N 1EH UK

**Keywords:** Tissue engineering, Esophagus, Esophageal stem cells

## Abstract

**Purpose:**

Long-gap esophageal atresia represents a significant challenge for pediatric surgeons and current surgical approaches are associated with significant morbidity. A tissue-engineered esophagus, comprising cells seeded onto a scaffold, represents a therapeutic alternative. In this study, we aimed to determine the optimal techniques for isolation and culture of mouse esophageal epithelial cells and to isolate CD34-positive esophageal epithelial stem cells from cadaveric mouse specimens.

**Methods:**

Primary epithelial cells were isolated from mouse esophagi by enzymatic dissociation from the mucosal layer (Dispase, Trypsin/EDTA) using three different protocols. In protocol A, isolated mucosa was minced and incubated with trypsin once. In protocol B, intact mucosal sheets underwent two trypsin incubations yielding a single-cell suspension. In protocol C, intact mucosa explants were plated epithelial side down. Epithelial cells were cultured on collagen-coated wells.

**Results:**

Initial findings showed that Protocol B gave the best results in terms of yield, viability, and least contamination with different cell types and microbes. Esophageal epithelial cells isolated using Protocol B were stained for CD34 and sorted using fluorescence-activated cell sorting (FACS). Of the total cells sorted, 8.3 % (2–11.3) [%median (range)] were CD34 positive.

**Conclusions:**

Our results demonstrate that mouse esophageal epithelial cells can be successfully isolated from fresh mouse esophagi using two consecutive trypsin incubations of intact mucosal sheets. Furthermore, the cells obtained using this method were successfully stained for CD34, a putative esophageal epithelial stem cell marker. Further research into the factors necessary for the successful proliferation of CD34 positive stem cell lines is needed to progress toward clinical application.

## Introduction

### Long-gap esophageal atresia

Esophageal atresia (EA) is a congenital malformation of the esophagus which presents after birth with a global incidence of between 1 in 2,500 and 1 in 5,000 live births [1, 2]. The precise mechanisms of causation of EA remain unknown; however, its development is thought to have its origin in endodermal proliferation in the 5th week of gestation.

Esophageal atresia is associated with tracheo-esophageal fistula (TEF) in almost 90 % of cases and, while the majority of cases are amenable to surgical anastomosis, serious complications include leakage from the anastomosis site, gastro-esophageal reflux, and a significant mortality rate [3]. Furthermore, for 10 % of EA cases, simple surgical anastomosis is not possible due to the presence of insufficient length in the distal segment of esophagus. These cases, where a TEF is not present, are known as long-gap EA and have required the development of new surgical approaches to repair, including circular myotomy [4] or interposition with colonic, gastric, or jejunal segments [5]. However, the non-mutually exclusive serious complications of leakage, stricture, and/or gastro-esophageal reflux remain a concern and lead to a mortality rate of 5.2 % [6]. Both EA and long-gap EA present significant challenges that are not fully addressed with current therapeutic options.

Tissue engineering (TE) is a multifaceted branch of regenerative medicine that combines expertise from cellular biology, molecular biology, and materials science to produce autologous constructs capable of replacing malformed, malfunctioning, or lost tissues and organs. Owing to the autologous sourcing of cells, TE addresses many of the issues which blight allotransplantation, such as the requirement for long-term immunosuppression and shortage of available donor organs.

**Fig. 1:**
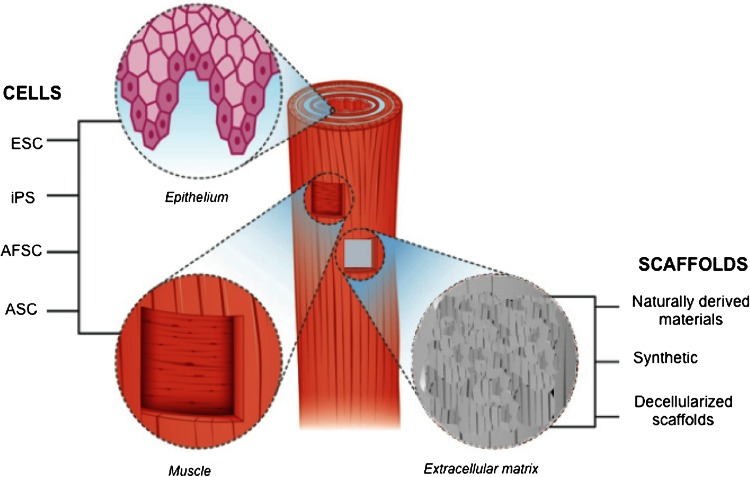
Esophageal TE requires the combination of appropriate scaffolds and cells. Cells used for repopulation of the epithelial and muscular layers can be derived from ESC, iPS, AFSC, and ASC; *ESC* embryonic stem cells, *iPS* induced pluripotent stem cells, *AFSC* amniotic fluid stem cells, *ASC* adult stem cells

Whole organ TE requires the union of two component parts, which when harmonized, accurately recreate the dynamic micro- and macro-environments of an organ structure, permitting both local function and global integration into the organism. These two core components are the patient’s own cells and an artificial synthetic or natural scaffold, onto which the cells are seeded (Fig. 1). Through the use of the above principles, TE has a promising potential to provide a therapeutic alternative to current surgical solutions for EA.

Tissue engineering has provided patients with autologous functional replacement tissue for a number of conditions, across a variety of clinical arenas to date [7]. TE has proven especially fruitful for hollow organs whose principal function is transit or storage. For example, four young male patients with traumatic damage to the urethra underwent urethral reconstruction with tissue-engineered urethral segments. These segments consisted of synthetic tubular scaffolds seeded with the patients’ muscle and epithelial cells. Three months after the surgical procedure, the four patients had achieved normal urine flow rates and normal histological structure without strictures in the reconstructed urethras [8]. Similar success has been achieved with tissue-engineered trachea, bronchus, bladder, and blood vessels [9–12].

In contrast to the success of TE when applied to the organs described above, tissue-engineered esophageal constructs have not been applied successfully in the clinical arena. However, preclinical studies have provided insights that may soon be translated for clinical use. Much of this preclinical work has highlighted the importance of the esophageal mucosal layer in preventing strictures in transplanted constructs. In an experiment to investigate the speed of epithelialization and viability of constructs after in vivo transplantation, Nakase et al. [13] compared seeded and non-seeded constructs. After 3 weeks, a mature epithelium was observed in the pre-seeded esophageal implants whereas the non-seeded controls showed reduced epithelialization and significant stricture formation. Furthermore, in the canine model, Badylak et al. demonstrated that esophageal constructs which had undergone specific ablation of the epithelium subsequently developed severe strictures when introduced into the in vivo environment [14]. These findings suggest that the luminal esophageal epithelium plays a key role in maintaining esophageal patency in both the native and artificial esophagus [15]. Further studies with acellular scaffolds have also reinforced the importance of the extra-luminal muscle layer of the esophagus for construct function. Yamamoto et al. [16, 17] transplanted acellular silicone tubes coated in a collagen ‘sponge’ into nine dogs and found that there was no infiltration of the construct with muscle cells at all time points up to a maximum of 26 months.

These findings from preclinical esophageal TE suggest important roles for both the epithelial cells of the esophagus and the external muscle layer, in recreating the functional esophagus with fidelity. Lack of either or both of these components appears to severely impair the functionality of constructs.

Isolation of esophageal epithelial cells has been attempted by several investigators to date; however, due to the diversity of isolation protocols used there is currently no single gold standard technique.

Early work focused on allowing cell migration from esophageal specimens onto cell culture plates following placement face down (i.e., explant culture) [18]. More recently, Kalabis et al. [19] have isolated whole mucosal sheets from Dispase-treated mouse esophagus that were then trypsinised and minced to obtain a cell suspension. Saxena et al. [20] used a different approach to isolate and culture esophageal epithelial cells from the rat. They used an isolation protocol whereby following overnight Dispase incubation and mucosal separation, the whole mucosa was incubated in trypsin–EDTA to dissociate individual cells [20].

The aim of this paper is to compare three of the most commonly used techniques for the isolation and effective culture of esophageal epithelial cells from mouse cadaveric specimens. After establishing the most effective technique of the three, we aim to further this protocol by isolating esophageal epithelial stem cells by applying known stem cell markers, principally CD34. The resulting population of CD34 positive cells represent a potential source of cells that may have great utility for the future TE efforts toward a replacement esophagus for patients with long-gap EA.

## Materials and methods

### Harvest of organs

All surgical procedures and animal husbandry were carried out in accordance with UK Home Office guidelines under the Animals (Scientific Procedures) Act 1986 and the local ethics committee. Adult C57/Bl-6 mice were euthanized by CO_2_ inhalation and cervical dislocation (*n* = 20). A midline incision was made to completely expose the abdominal and thoracic cavities. The esophagus was harvested from the cervical portion to the gastro-esophageal junction and washed with phosphate buffered solution containing 1 % solution of antimycotic and antibiotic (PBS/AA; Sigma, UK).

### Cell isolation

#### Epithelium, protocol A

Freshly isolated mouse esophagi were cut longitudinally to expose mucosa and rinsed with PBS/AA. Esophagi were incubated with 1 U/mL Dispase-I (Roche, UK) and 1 % AA for 15 min at 37 °C, following which, the mucosal sheets were separated from the submucosa and finely minced using a scalpel (BD Biosciences, UK).The minced mucosal tissue was incubated with 0.05 % trypsin/EDTA (Sigma, UK) and 1 % AA at 37 °C for 10 min. An equal volume of 200 μg/mL soybean trypsin inhibitor (Sigma, UK) was added. The epithelial cell suspension was passed through 40 μm cell strainer (BD Biosciences, UK), centrifuged at 1,500 rpm for 5 min at 4 °C, and the pellet was resuspended in keratinocyte serum-free medium supplemented (K-SFM^+^; Life Technologies, UK) with 1 ng/mL Epidermal Growth Factor (EGF; Sigma, UK) 0.4 % Bovine Pituitary Extract (BPE; Sigma, UK), 2 % Fetal Bovine Serum (FBS; Sigma, UK) 10 mM HEPES (Sigma, UK) and 1 % AA. Isolated cells were seeded onto 6-well plates and maintained at 37 °C, 5 % CO_2_. The plates were either freshly coated with rat tail collagen I (BD Biosciences, UK), coated and then air-dried for 24 h or not coated at all. The medium was changed every 2–3 days and cells were passaged when they reached 60 % confluence.

#### Epithelium, protocol B

Freshly isolated mouse esophagi were cut longitudinally and rinsed with PBS/AA. Esophagi were incubated with 1 U/mL dispase-I and 1 % AA for 15 min at 37 °C, following which, the mucosal sheets were separated from the submucosa, pooled into 1 mL 0.05 % trypsin–EDTA and 1 % AA, incubated at 37 °C for 10 min and vortexed for 10 s. The supernatant was removed and pipetted into 8 mL of soybean trypsin inhibitor (250 μg/mL in PBS). To examine the need for further isolation, the remaining mucosa was incubated in another 1 mL trypsin-EDTA and 1 % AA to 37 °C for 5 min, and the supernatant added to 8 mL of Soybean trypsin inhibitor. The resulting cell suspensions were filtered separately through a 40 μm cell strainer and centrifuged at 188*g* for 5 min at 4 °C. The pellets were resuspended in K-SFM^+^ medium. Isolated cells were seeded onto 6-well collagen-coated plates and maintained at 37 °C, 5 % CO_2_. The medium was changed every 2–3 days and cells were passaged when they reached 60 % confluence.

#### Epithelium, protocol C

Freshly isolated mouse esophagi were cut longitudinally to expose mucosa and rinsed with PBS/AA. Esophagi were incubated with 1 U/mL Dispase-I and 1 % AA for 15 min at 37 °C, following which, the mucosal sheets were separated from the submucosa and incubated with 0.05 % trypsin/EDTA and 1 % AA at 37 °C for 10 min. Following the trypsin treatment, mucosal sheets were placed into HBSS (Sigma, UK) and 1 % AA, and cut into 5 mm segments using a sterile scalpel. The segments were placed mucosal surface face down onto rat tail collagen-1 (BD Biosciences) coated wells of a 6-well plate. Explants were left to attach for 20 min, at which point K-SFM^+^ medium was added to cultures. After 24 h, explants were removed from the wells. The medium was changed every 2–3 days and cells were passaged when they reached 60 % confluence.

#### Bone marrow

Mouse bone marrow (BM) was isolated from two hind limb bones of C57/Bl-6 mice. A 27G (BD Biosciences, UK) 1 mL needle syringe was pushed into the bone cavity and the BM was flushed out with 1 mL PBS. The bone marrow was filtered through a 70 μm cell strainer (BD Biosciences, UK). The resulting cell suspension was centrifuged at 188*g* for 5 min at 4°C and kept on ice.

### Fluorescence-activated cell sorting (FACS)

Primary esophageal epithelial cells were isolated using the protocol described in “Epithelium, protocol B”. Following centrifugation, the cell pellet was resuspended in 200 μL of staining buffer (SB; PBS, pH 7.2, 0.5 % bovine serum albumin [BSA; Sigma, UK], and 2 mM EDTA). For the unstained control, 20 μL of the cell suspension was removed and 80 μL of SB buffer was added to it. The remaining 180 μL of cell suspension was stained with PE Rat anti-Mouse CD34 antibody. The cells were stained using an antibody concentration of 1:100 at 4 °C for 30 min. The cells were then washed with 1 ml of SB buffer and centrifuged at 300×*g* for 5 min at 4 °C. The cell pellet was resuspended in 500 μL of SB buffer. Freshly isolated bone marrow cells were used as a positive control for CD34. Following isolation the pellet was resuspended in 200 μL of SB buffer, 150 μL of which was stained with CD34 PE (BM^+^ control), and 50 μL of which was not stained (BM^−^ control). Staining with the Viaprobe [7-amino-actinomycin D (7-AAD)] was used to exclude dead cells. Flow cytometry was performed on a MoFlo XDP cell sorter.

### Immunofluorescence

Cultures were fixed in 4 % paraformaldehyde (PFA) for 15–20 min at room temperature and washed twice (5 min each) with 1 × Rinse Buffer [PBS/Tween 20 (0.05 %); Sigma, UK]. Permeabilization was achieved with 0.1 % Triton X-100/PBS (Sigma, UK) for 10 min at room temperature (RT). Cultures were washed in PBS for 5 min three times. Blocking solution (4 % goat serum/PBS; Sigma, UK) was applied for 30 min at RT. Primary antibody incubation was performed using pan-cytokeratin (Rabbit polyclonal; Abcam, UK) (dilution 1:100) overnight at 4 °C. Cells were washed for 5 min, three times with 1X Rinse Buffer. Cells were incubated with secondary antibody (Alexa-Fluor 488) for 60 min at RT and washed three times (5 min each) with 1 × Rinse Buffer. Nuclei were counterstained with 4′,6-diamidino-2-phenylindole (DAPI; dilution 1:1,000) and Sudan Black was used to quench autofluorescence. Images were captured using Axio-plot2 Carl Zeiss fluorescence microscope.

### CD34^+^ fraction culture

10 × 10^4^ Swiss 3T3 fibroblasts were plated onto each well of a 6-well plate in DMEM/10 %FBS (Sigma, UK) and incubated at 37 °C, 5 % CO_2_ for 24 h to allow attachment. To mitotically inactivate the fibroblasts, medium was replaced with 2.5 mL of 10 μg/mL Mitomycin C (Sigma, UK), incubated for 1 h and washed three times with PBS, following which 2 mL of William’s E medium/20 % FBS (Invitrogen, UK) was placed in each well. Following sorting using the MoFlo XDP cell sorter the CD34 positive fraction was seeded on the mitotically inactivated Swiss 3T3 fibroblast feeder layers.

### Rhodamine B colony formation assay

CD34^+^ sorted cells were maintained in culture for 2 weeks, after which medium was removed and 4 % paraformaldehyde (PFA) was applied to fix the cells. After 10 min at room temperature, cultures were washed with PBS and colonies were stained with 1 % Rhodamine B solution, for 20 min, and then washed with water. Colonies were visualized under light microscopy.

## Results

Early experiments focused on optimization of cell isolation steps that varied greatly across the literature. For example, bacterial contamination was avoided by supplementing all protocol steps with 1 % AA. Dispase-I concentration was tested across the range of 0.1–10 U/mL, with 1 U/mL showing the best effect in separating the mucosa while preserving the overall structure. Medium change following isolation was shown to allow the attachment of a greater number of cells if done at 72 h rather than 24 h (data not shown).

**Fig. 2:**
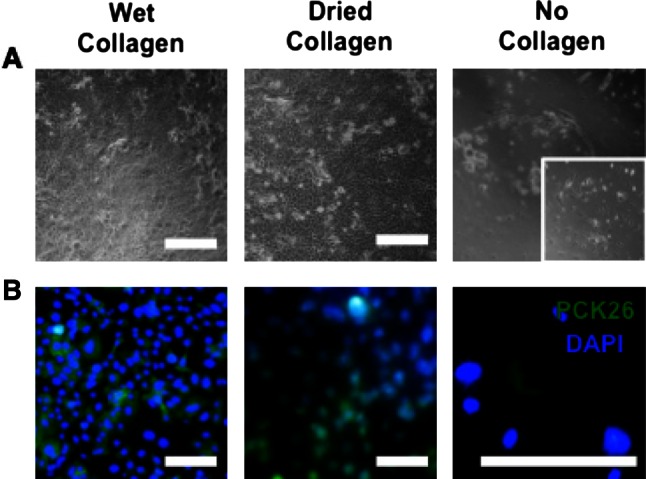
Collagen coating of plates (wet or dried) allowed a higher number of isolated cells to attach and grow following protocol A as shown by the light microscopy images (**a**). In the wells with no collagen spindle-shaped cells were mostly seen (*inset*). Pan-cytokeratin staining for epithelial cells demonstrated the epithelial identity of cells seeded onto collagen-coated plates, with no positive staining in the lack of collagen (**b**); *scale bar* 100 μm

Mouse esophageal epithelial cells were isolated using protocol A and plated onto either freshly collagen-coated wells, collagen-coated wells that were air-dried for 24 h, or wells with no coating. Cells were seen to attach in similar numbers onto freshly coated and air-dried collagen wells. Epithelial cell monolayers, with distinctive cobblestone morphology, were observed in the fresh and dried collagen-coated wells after 3 weeks, whereas no attachment of cells was observed in the non-collagen-coated plates (Fig. 2a). In wells lacking collagen coating spindle-shaped cells were seen attaching during the first 72 h (Fig. 2a, inset) that mostly died off by 1 week. Cultures were characterized by immunofluorescence to ensure true epithelial identity. Cultures were fixed and stained with anti-mouse pan-cytokeratin 26 (PCK-26) antibody, with DAPI used as a fluorescent nuclear counterstain. Epithelial cell cultures on collagen-coated wells showed positivity for PCK-26 (Fig. 2b). There was very little PCK-26 positivity present in the non-collagen plate (Fig. 2b). In all isolation with protocol A, there was significant contamination with fibroblasts (Fig. 2a, inset).

**Fig. 3:**
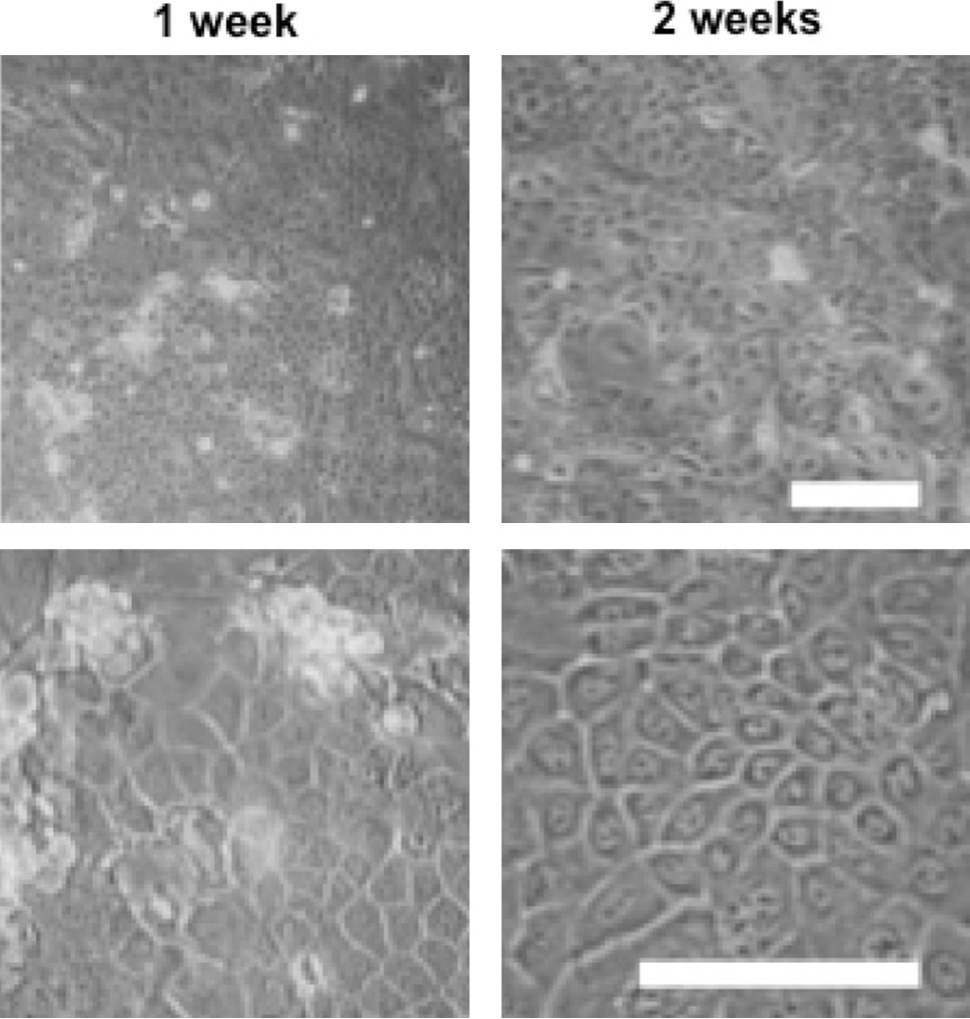
Cells isolated using protocol B showed a characteristic epithelial cobblestone morphology in culture (**a**), forming a monolayer by 2 weeks (**b**); *scale bar* 100 μm

The isolation and culture of esophageal epithelial cells using protocol B successfully yielded epithelial cell cultures. Cells were cultured on freshly collagen-coated wells and epithelial cell colonies were visible within 1 week of plating, which had resulted in confluent epithelial monolayer by 2 weeks (Fig. 3). Isolation with the first trypsin incubation was successful in all experiments (8/8) and the use of a second trypsin incubation did not isolate further cells (data not shown).

**Fig. 4:**
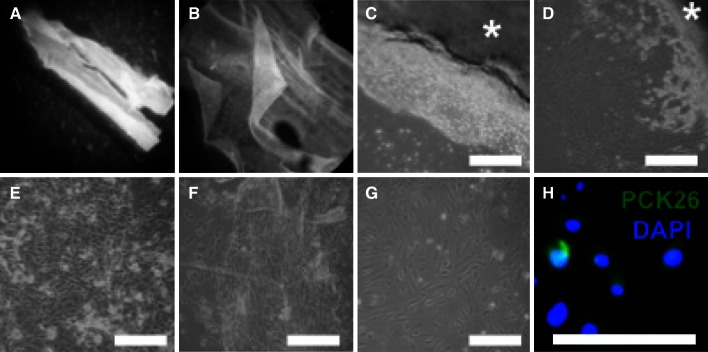
In protocol C, whole mucosal sheets were isolated (**a**), the mucosal side was identified by the mucosal ridges (**b**) and plated. Cells were seen migrating out of the mucosal sheet (**c**, *asterisk*) and acquiring cobblestone morphology (**d**). In 33 % of isolations, epithelial colonies were identified after 6 days (**e**), while 67 % of isolations were unsuccessful in doing so (**f**). In all cases, there was concomitant migration and attachment of spindle-shaped cells that did not stain positive for PCK-26 (**g**, **h**); *scale bar* 100 μm

For protocol C (i.e., explant technique) whole mucosal sheets were isolated (Fig. 4a) and the mucosal side was confirmed by observing the mucosal ridges under microscope (Fig. 4b) that were then placed face down on tissue culture plates coated with collagen. Under a cell culture microscope, the cells can be seen migrating out of the mucosal sheet (Fig. 4c, asterisk) and forming the distinctive cobblestone morphology (Fig. 4d).The majority of explants detached easily from culture plates upon the addition of medium. Culture outcomes, however, were very variable. In 33 %, (3/9) of isolations areas of epithelial cell confluence were identified after 6 days (Fig. 4e). In 67 %, (6/9) of isolations explant cultures did not generate viable epithelial cell cultures with differentiated epithelial squames attaching and many cells remaining unattached and suspended in the medium (Fig. 4f). In all cases, there was concomitant migration and attachment of spindle-shaped cells that did not stain positive for PCK-26 (Fig. 4g, h). Immunofluorescence characterization of explant cultures in general showed weak positivity for PCK-26 (Fig. 4h).

**Fig. 5:**
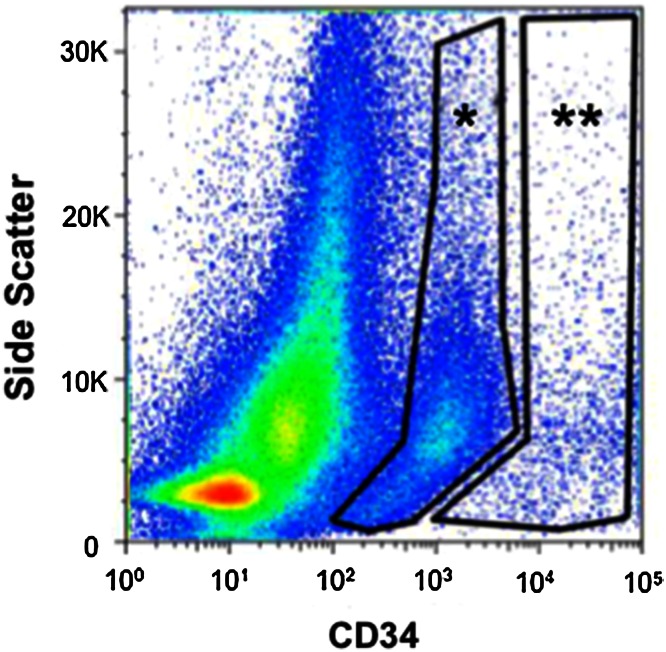
CD34 FACS sorting gates for CD34 *dim* (*) and *bright* (**) populations

**Fig. 6:**
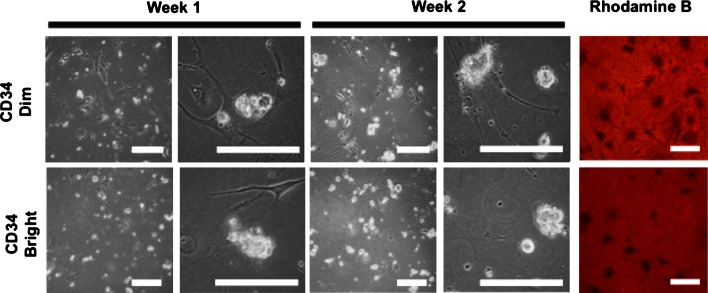
CD34^+^esophageal epithelial cells were sorted onto mitotically inactivated Swiss 3T3 fibroblasts. *Dim* and *bright* subpopulations showed similar growth characteristics in vitro, with Rhodamine B staining confirming colony formation at 2 weeks; *scale bar* 100 μm

Esophageal epithelial cells isolated using protocol B were analyzed by FACS for CD34 positivity, a putative stem cell marker. Different gates were placed subdividing the fluorescence of positively staining cells into brightly fluorescent and dimly fluorescent populations. A characteristic FACS sort gate is shown in Fig. 5. An average mean percentage of the CD34^+^ bright population constituted 1.6 %, whereas the CD34^+^ Dim population constituted 9.2 % of total sorted cells (Table 1). The CD34^+^ cells were directly sorted onto irradiated Swiss 3T3 fibroblasts and cultured for up to 2 weeks. CD34^+^ dim and bright subpopulations demonstrated similar growth characteristics in culture (Fig. 6). Staining with Rhodamine B to check for colony formation was positive after 2 weeks (Fig. 6, Rhodamine B).

**Table 1:** Percentage of total sorted CD34^+^ cells in each subpopulation; median (range)

Cells	CD34^+^ (%)
Esophageal epithelial cells	8.3 (2–11.7)
CD34^+^ bright subpopulation	1.6 (1.1–1.7)
CD34^+^ dim subpopulation	9.2 (4.8–10)
Unstained control	0.05 (0.04–0.06)

## Discussion

Esophageal TE could provide a vitally needed alternative for the repair of long-gap EA, which still poses a significant challenge to pediatric surgeons, and could be used for other esophageal disease of children and adults. The seeding of autologous cells onto a decellularized esophageal cadaveric scaffold could lead to the development of functional esophageal tissue. A principal requirement to achieve this is successful isolation and culture of esophageal epithelial cells. We set out to identify the optimal method for isolating esophageal epithelial cells by comparing and fine-tuning previously reported protocols. We also assessed whether collagen-coated plates improve epithelial cell proliferation in vitro, and finally, we aimed to assess the possibility of isolating esophageal epithelial stem cells using CD34 as a stem cell marker.

The results of this study show much concordance with previous efforts in culturing mouse esophageal epithelial cells. In the rat, Beckstead and colleagues investigated whether the adhesion of esophageal epithelial cells was affected by coating of culture wells. They compared groups of cells cultured in wells coated with collagen I, collagen IV, fibronectin, laminin, and osteopontin both between groups and of each group to a non-coated control group [21]. They found that all of the tested coatings increased adhesion of cells, while the two collagen groups showed the highest rates of adhesion. In the mouse model, Kalabis et al. [19] explored the use of enriched collagen gels containing esophageal fibroblasts or a dual layer of collagen underneath a collagen–fibroblast–Matrigel composite [22]. Both of these culture environments used collagen as part of a 3D organotypic culture system. Similarly, Saxena et al. [20] cultured esophageal epithelial cells on a prefabricated porcine collagen–elastin scaffold.

Therefore, the results of the present study on the influence of collagen coating on the adhesion of esophageal epithelial cells in culture appear to be concordant with the previous findings of other investigators. Furthermore, on the question of whether fresh or air-dried collagen coatings are superior, no significant difference was demonstrated.

Good isolation of epithelial cells was achieved using both whole esophageal mucosa and minced tissue. However, the latter technique introduced contamination by esophageal fibroblasts. This finding may be explained by considering the mechanical effect of mincing on the esophageal mucosa; it increases the surface area of the mucosal tissue, thereby exposing a greater proportion of intercellular junctions to the dissociative actions of trypsin and EDTA. Furthermore, it allows the reagents to work on a greater area of the submucosa, which will allow the release of more stromal cells when compared to a non-mincing protocol. For this reason, it is perhaps unsurprising that mincing introduced a level of fibroblast contamination that was not seen with trypsinization of whole mucosa.

Although repeating trypsinization was not shown to increase the isolation of cells from the esophageal mucosa specimens, the addition of further techniques used in other investigations may help to increase isolation yield in future studies. Previous groups have employed a stage of vortexing [20, 23] or constant pipetting [21] during the incubation with trypsin to further the disruption of cell–cell adhesions and thereby to increase the yield of single cells. The successful application of these additional techniques will be required to maximize the eventual clinical applicability of the findings described herein.

Culture using mucosal explants (protocol C) showed variable results with unreliable cell attachment and growth, along with fibroblast contamination. With consideration given to the migratory characteristics of fibroblasts, it appears logical that cultures resulting from the application of protocol C suffered a certain degree of fibroblast contamination. The results of the present study appear at odds with a previous application of the explant technique for culturing esophageal epithelial cells [2], which found minimal contamination of the cell culture with fibroblasts. The contrasting results of this previous work on explant culture may be explained by their use of a rat model; however, the contribution of this difference in experimental design cannot be completely quantified.

Cells isolated with protocol B that expressed CD34 produced colonies after 2 weeks in vitro. Our finding that (both dim and bright) populations of CD34^+^ cells formed colonies within 2 weeks when cultured on fibroblast feeder layers provides further support for the role of CD34 as a putative marker of esophageal epithelial stem cells. Unexpectedly, both dim and bright populations displayed similar growth characteristics in culture. This suggests that further work is necessary to fully elaborate the implications of CD34 positivity in esophageal epithelial stem cells.

In this study, we have optimized an isolation protocol for esophageal epithelial cells from mouse tissue. To further our work, the protocol needs to be examined in the human model using small biopsies as a source, to mirror the process that will need to be followed for clinical autologous use. Seeding of the isolated epithelial cells on natural or synthetic scaffolds in bioreactor environments represents the next step toward a tissue-engineered esophageal replacement.
